# Comprehensive analysis of the *ATM*, *CHEK2 *and *ERBB2 *genes in relation to breast tumour characteristics and survival: a population-based case-control and follow-up study

**DOI:** 10.1186/bcr1623

**Published:** 2006-11-28

**Authors:** Kristjana Einarsdóttir, Lena U Rosenberg, Keith Humphreys, Carine Bonnard, Juni Palmgren, Yuqing Li, Yi Li, Kee S Chia, Edison T Liu, Per Hall, Jianjun Liu, Sara Wedrén

**Affiliations:** 1 Department of Medical Epidemiology and Biostatistics, Karolinska Institute, Nobels väg 12A, 171 77 Solna, Sweden; 2 Population Genetics, Genome Institute of Singapore, 60 Biopolis Street, Genome #02-01, Singapore 138672; 3 Department of Mathematics, Stockholm University, Roslagsvägen 101, 106 91 Stockholm, Sweden; 4 Information and Mathematical Sciences, Genome Institute of Singapore, 60 Biopolis Street, Genome #02-01, Singapore 138672; 5 Centre for Molecular Epidemiology, Department of Community, Occupational and Family Medicine, National University of Singapore, 16 Medical Drive, Singapore 117597

## Abstract

**Background:**

Mutations in the ataxia-telangiectasia mutated (*ATM*) and checkpoint kinase 2 (*CHEK2*) genes and amplification of the v-erb-b2 avian erythroblastic leukemia viral oncogene homolog 2 (*ERBB2*) gene have been suggested to have an important role in breast cancer aetiology. However, whether common variation in these genes has a role in the development of breast cancer or breast cancer survival in humans is still not clear.

**Methods:**

We performed a comprehensive haplotype analysis of the *ATM*, *CHEK2 *and *ERBB2 *genes in a Swedish population-based study, which included 1,579 breast cancer cases and 1,516 controls. We followed the cases for 8.5 years, on average, and retrieved information on the date and cause of death during that period from the nationwide Swedish causes of death registry. We selected seven haplotype-tagging SNPs (tagSNPs) in the *ATM *gene, six tagSNPs in the *CHEK2 *gene and seven tagSNPs in the *ERBB2 *gene that predicted both haplotypic and single locus variations in the respective genes with *R*^2 ^values ≥ 0.8. These tagSNPs were genotyped in the complete set of cases and controls. We computed expected haplotype dosages of the tagSNP haplotypes and included the dosages as explanatory variables in Cox proportional hazards or logistic regression models.

**Results:**

We found no association between any genetic variation in the *ATM*, *CHEK2 *or *ERBB2 *genes and breast cancer survival or the risk of developing tumours with certain characteristics.

**Conclusion:**

Our results indicate that common variants in the *ATM*, *CHEK2 *or *ERBB2 *genes are not involved in modifying breast cancer survival or the risk of tumour-characteristic-defined breast cancer.

## Introduction

Twin and family studies consistently indicate that the risk of breast cancer is influenced, in part, by hereditary factors, but high-risk mutations seem to account for only 1–2% of all breast cancer cases in the general population [1]. A polygenic model has been proposed to account for the residual familial risk [2], which anticipates small effects of several low-penetrance genetic risk variants in combination with environmental influence. Although no human data exist for the affect of germ-line polymorphisms on tumour outcome, there is strong evidence that strain background is a significant determinant of the clinical behaviour of experimental mammary carcinomas in mice [3-5]. We thus set out to study the role of common variation in key breast cancer candidate genes in relation to breast cancer aetiology, survival and tumour characteristics.

The ataxia-telangiectasia mutated (*ATM*; MIM 607585), checkpoint kinase 2 (*CHEK2*; MIM 604373) and v-erb-b2 avian erythroblastic leukemia viral oncogene homolog 2 (*ERBB2*; also named *HER2*; MIM 164870) genes have been suggested to have an important role in breast cancer aetiology. The ATM protein is activated in response to ionizing radiation and triggers phosphorylation of CHEK2 and other proteins that promote cell cycle arrest and activation of DNA repair [6-12]. The *ATM *gene is mutated in the rare autosomal recessive disorder ataxia-telangiectasia (A-T) and the risk of breast cancer has been found to be increased in relatives of A-T patients [13,14], in addition to A-T heterozygotes [15,16]. Mutations in the *CHEK2 *gene have been found in patients with Li-Fraumeni syndrome [17] and one such mutation – the rare *1100delC *gene mutation – has been found to increase breast cancer susceptibility at the population level [18] and in families without *BRCA1 *or *BRCA2 *gene mutations [19,20]. ERBB2 is a transmembrane glycoprotein, with tyrosine kinase activity [21-25], that has a major role in signal transduction, thereby affecting cell proliferation, differentiation, motility and survival [26-28]. The *ERBB2 *gene is amplified and/or overexpressed in approximately 30% of breast tumours, a phenomenon that is associated with a poor prognosis [29-31].

Until now, common variation in the *ATM, CHEK2 *and *ERBB2 *genes has mainly been studied in relation to the overall risk of breast cancer, but the results have been inconclusive. It is worthwhile studying common variation in the *ATM*, *CHEK2 *and *ERBB2 *genes in relation to breast cancer progression because defects in the genes could increase the risk of developing tumours with an unfavourable prognosis through their role in the regulation of cell cycle checkpoints and amplification. Variation in the genes could also affect cancer survival through increased radiosensitivity [32-34]. One group found a relationship between poor breast cancer prognosis and common haplotypes in the *ERBB2 *gene [35], but to our knowledge, nothing has been reported regarding the association between common haplotypes in the *ATM *and *CHEK2 *genes and breast cancer survival or tumour characteristics. Hence, many questions regarding the role of these genes in breast cancer survival and the progression of breast cancers are unanswered.

We performed a haplotype analysis of the *ATM*, *CHEK2 *and *ERBB2 *genes by genotyping a dense set of markers in each gene in 92 randomly selected controls, thus acquiring a comprehensive coverage of the common variation in each entire gene. We genotyped selected haplotype-tagging SNPs (tagSNPs) in a well-defined, Swedish population. We then assessed the association of the tagSNPs in the *ATM*, *CHEK2 *and *ERBB2 *genes and their haplotypes with breast cancer survival and the risk of tumour-characteristic-defined breast cancer. We also studied the tagSNPs and haplotypes in the *ATM *and *ERBB2 *genes, in addition to two mis-sense mutations in the *ATM *gene (2572 T→C and 4258 C→T), in relation to the overall risk of breast cancer.

## Materials and methods

### Study population

The study base included all Swedish-born women between 50 and 74 years of age who were resident in Sweden between October 1993 and March 1995. During that period, we identified all breast cancer cases at diagnosis through the six regional cancer registries in Sweden, which provide virtually complete data on incident cancers in Sweden [36]. We randomly selected controls, who matched the cases in 5-year age strata, from the Swedish registry of the total population. Of the eligible women, 3,345 (84%) breast cancer cases and 3,454 (82%) controls participated in this initial questionnaire-based study, providing detailed information on their use of menopausal hormone therapy, their reproductive history and other lifestyle factors. Results from the study have been published [37-41].

From this initial study, we randomly selected 1,500 breast cancer cases and 1,500 age–frequency-matched controls among the postmenopausal participants without any previous malignancy (except carcinoma *in situ *of the cervix or nonmelanoma skin cancer). With the intention of increasing the statistical power in subgroup analyses, we further selected all of the remaining breast cancer cases and controls who had used menopausal hormones (oestrogen alone or any combination of oestrogen and progestin) for at least 4 years (191 cases and 108 controls) and all women with self-reported diabetes mellitus (110 cases and 104 controls). Additionally, 345 controls, who were included in both the initial breast cancer study and an endometrial cancer study with the same study base and inclusion criteria, were added to the control group. In total, we selected 1,801 breast cancer cases and 2,057 controls.

After informed consent was obtained, participants donated whole blood. For deceased breast cancer patients and women who declined to donate blood but consented to the use of tissue samples, we collected archived paraffin-embedded, noncancerous tissue samples. We acquired 70% of the requested tissue samples; the main reason for nonparticipation was unwillingness or a lack of time at the respective pathology department to provide the tissue blocks. In total, we obtained 1,321 blood samples and 275 archived tissue samples from the breast cancer patients and 1,524 blood samples from the controls. The mean time from diagnosis to the arrival of the blood and tissue samples at our department was 5 years. Reasons for nonparticipation included a lack of interest in research, a negative attitude towards genetic research, old age and severe disease or death. Population-based participation rates (taking into account the proportion of individuals who did not participate in the questionnaire-based study) for the breast cancer cases and controls were 75% and 61%, respectively.

This study was approved by the Institutional Review Boards in Sweden and the National University of Singapore.

### DNA isolation

The Swegene laboratories in Malmö (Sweden) extracted DNA from 4 ml of whole blood using the QIAamp DNA Blood Maxi Kit (Qiagen, Solna, Sweden) according to the manufacturer's instructions. From nonmalignant cells in paraffin-embedded tissue, we extracted DNA using a standard phenol/chloroform/isoamyl alcohol protocol [42]. We successfully isolated DNA from 1,318 blood samples and 272 tissue samples from the breast cancer patients and 1,518 blood samples from the controls.

### SNP markers and genotyping

The *ATM *gene covers 146.3 kb of genomic sequence on chromosome 11, the *CHEK2 *gene spans 54.1 kb on chromosome 22 and the *ERBB2 *gene covers 33.7 kb on chromosome 17 (build 125 in the dbSNP (Single Nucleotide Polymorphism database). We selected SNPs in the *ATM*, *CHEK2 *and *ERBB2 *genes and their 10 kb flanking sequences from dbSNP (build 124) and Celera databases aiming for an initial marker density of at least one SNP per 5 kb. SNPs were genotyped using the Sequenom primer extension-based assay (San Diego, CA, USA) and the BeadArray system (Illumina, San Diego, CA, USA) according to the manufacturers' instructions. All genotyping results were generated and checked by laboratory staff unaware of case–control status. Only SNPs for which >85% of the samples gave a genotype call were analysed further. As a quality control, we genotyped 200 randomly selected SNPs (not including SNPs in the *ATM*, *CHEK2 *or *ERBB2 *genes) in the 92 control samples using both the Sequenom system and the BeadArray system. The genotype concordance was >99.5%, suggesting high genotyping accuracy.

### Characterization of linkage disequilibrium and haplotype-tagging SNP selection

We produced linkage disequilibrium (LD) plots of the D' values for *ATM *and *ERBB2 *genes (supplementary Figures 1 and 2, respectively) using the Haploview program [43]. We reconstructed haplotypes for all three genes using the partition-ligation-expectation-maximization (PLEM) algorithm [44] implemented in the tagSNPs program [45] and selected tagSNPs according to the *R*^2 ^coefficient, which quantifies how well the tagSNP haplotypes predict the SNPs or the number of copies of haplotypes that an individual carries. We chose tagSNPs so that common SNP genotypes (minor allele frequency ≥0.03) and common haplotypes (frequency ≥0.03) were predicted by *R*^2 ^values ≥ 0.8 [46]. To evaluate the performance of our tagSNPs in capturing unobserved SNPs within the genes and to assess whether a denser set of markers was needed, we performed a SNP-dropping analysis [47,48]. In brief, each of the genotyped SNPs was dropped in turn and tagSNPs were selected from the remaining SNPs so that their haplotypes predicted the remaining SNPs with an *R*^2 ^value of 0.85. We then estimated how well the tagSNP haplotypes of the remaining SNPs predicted the dropped SNP, an evaluation that can provide an unbiased and accurate estimate of tagSNP performance [47,48].

### Tumour characteristics and follow-up

We retrieved information on the date and cause of death (until 31 December 2003) from the Swedish causes of death registry and the date of emigration from the Swedish national population registry. Information from the causes of death registry in Sweden has been found to be of high quality [49]. The follow-up time began at the date of diagnosis and ended on 31 December 2003 or at the date of death or emigration, whichever came first. We collected information on tumour characteristics, such as tumour size, lymph-node involvement, grade (tumour differentiation), histological type and date of the first distant metastasis, from medical records. We obtained information on the oestrogen and progesterone receptor content and S-phase fraction (i.e. the proportion of tumour cells in the DNA synthesis phase of the cell cycle) of the tumours from seven laboratories around Sweden that routinely perform these tumour measurements for the whole country. At the time of the study, all seven laboratories used an enzyme immunoassay (Abbott Laboratories, Solna, Sweden) on cytosol samples for analysing the oestrogen and progesterone receptor content. This method was oestrogen receptor type α specific [50]. The laboratories reported either quantitative measurements (fmol receptor per μg DNA or mg protein and the percentage of cells in S-phase) or categorical measurements (strongly positive, positive, weakly positive or negative for receptor status and high, intermediate or low S-phase fraction). A rather high proportion of this information was missing, which was owing to the fact that these measurements were not routinely performed in the mid-1990s. We classified the tumour characteristics as follows:

1. TNM stage:

1.a. Stage 1 – tumour size ≤20 mm and no regional lymph-node metastases.

1.b. Stage 2 – tumour size ≤20 mm and lymph-node metastases, or tumour size 20–≤50 mm, or tumour size >50 mm and no lymph-node metastases.

1.c. Stage 3 – an inflammatory breast tumour, or tumour size >50 mm and lymph-node metastases.

1.d. Stage 4 – distant metastasis within 90 days of diagnosis.

2. Lymph-node involvement:

2.a. Yes – at least one metastasised lymph node.

2.b. No – no metastasised lymph node.

3. Grade:

3.a. High differentiation.

3.b. Intermediate differentiation.

3.c. Low differentiation.

4. Oestrogen and progesterone receptor status:

4.a. Positive – ≥0.05 fmol/μg DNA, ≥10 fmol/mg protein or categorically strongly positive, weakly positive or positive.

4.b. Negative – <0.05 fmol/μg DNA, <10 fmol/mg protein or categorically negative.

5. S-phase fraction:

5.a. High – ≥9% or categorically high.

5.b. Low – <9% or categorically low.

We combined TNM stage 3 and TNM stage 4 in all association analyses because of small numbers.

### Statistical analyses

In assessing the association with tumour-characteristic-defined breast cancer, we stratified the cases on breast cancer subtypes and compared each group with the controls. Our testing strategy was to fit a single model and assess within each stratum of risk-factor subgroup and for different tumour characteristics, haplotype-trait associations as a global likelihood ratio test [51]. We first computed expected haplotype dosage using the tagSNPs program [45], with haplotype frequencies estimated for the breast cancer cases and controls combined, assuming Hardy–Weinberg equilibrium (HWE) of haplotypes. We then, included the haplotype dosages as explanatory variables in the regression models. To estimate the power in the risk component of the study, we used a method described by Chapman and colleagues [52], which assumes co-dominant effects at an unobserved locus. To calculate the power for log-additive effects in the survival component of the study, we used the Quanto program [53] in a similar manner to that described by Manolio and colleagues [54].

We applied unconditional logistic regression models adjusted for age (in 5-year age-groups) to assess the relationship between the *ATM*, *CHEK2 *and *ERBB2 *tagSNP haplotypes and the overall risk of breast cancer, in addition to breast cancer subtypes. We estimated the hazard ratio of death owing to breast cancer in relation to the genes' tagSNP haplotypes using Cox proportional hazards models. The appropriateness of these approaches is supported by Stram and colleagues [45]. That is, if *R*^2 ^values are high, such as here, the point and interval estimates obtained by this approach will be approximately accurate. To assess the proportional hazards assumptions of the Cox models, we examined scaled Schoenfeld residuals and found no evidence against proportionality.

'Confounding' has been defined as the presence of a common cause of the exposure and outcome [55]. We believe that lifestyle and reproductive breast cancer risk factors are unlikely to cause genetic variation in the genes, but they could be intermediates in the causal pathway between the genes and both breast cancer and tumour-characteristic-defined breast cancer. For completeness, we assessed whether the tagSNPs were associated with known breast cancer risk factors (age at menarche, age at menopause, body mass index, age at first birth, parity, menopausal hormone use and diabetes mellitus) among the randomly selected controls using Kruskal-Wallis and Chi square tests. Analyses were performed using the SAS system (release 9.1; SAS Institute Inc., Cary, NC, USA).

## Results

### Study population

Characteristics of breast cancer cases in the parent case–control study and the current genetic association study are described in Table 1. With respect to nongenetic factors among cases and controls [56] and tumour characteristics among cases (Table 1), the current study was representative of the parent study. Breast-cancer-specific death rates among the cases in the current study reflected known prognostic information on various tumour characteristics (Table 1). Tumours with unknown hormone receptor status or S-phase fraction were smaller in both parent and current studies (*P *< 0.0001 for both receptor status and S-phase fraction). Missing information on tumour grade depended significantly on tumour size in the parent study (*P *= 0.01) but not in the current study (*P *= 0.15).

Cases who participated through tissue sample donation were, on average, 1.5 years older (*P *= 0.0003) and more likely to have been diagnosed with TNM stage 2 or more advanced cancers (*P *< 0.0001) compared with cases who donated a blood sample. Importantly, no significant differences in the genotype frequencies were evident between cases who participated through blood or tissue sample donation.

### Estimation of the pattern of linkage disequilibrium and coverage

We summarize the statistics for the genotyping results and SNP coverage in the *ATM*, *CHEK2 *and *ERBB2 *genes in supplementary Table 1. The SNPs in the *ATM*, *ERBB2 *and *CHEK2 *genes that were successfully genotyped in 92 randomly selected controls are listed in supplementary Table 2 (*ATM*) and 3 (*ERBB2*), and have been previously published in Table 2 by Einarsdóttir and colleagues [56] (*CHEK2*). In brief, our study included 51 SNPs in the *ATM *gene, 14 SNPs in the *CHEK2 *gene and 13 SNPs in the *ERBB2 *gene that were successfully genotyped – and were in HWE – in the 92 controls. The mean spacing between the SNPs included in our study was 2.9 kb in the *ATM *gene, 4.0 kb in the *CHEK2 *gene and 2.8 kb in the *ERBB2 *gene. We detected strong LD across all three genes [supplementary Figure 1, Figure 1 in Einarsdóttir and colleagues [56] and supplementary figure 2, respectively]. Using the SNP dropping method [47], we found that the tagSNPs selected from the SNPs included in our study could capture nongenotyped SNPs as efficiently as the SNPs included in our study (supplementary Table 1).

### Breast cancer survival

Table 2 summarizes the information on the tagSNPs in the *ATM, CHEK2 *and *ERBB2 *genes that we genotyped in breast cancer cases and controls. All of the tagSNPs in the *CHEK2 *and *ERBB2 *genes and two of the tagSNPs in the *ATM *gene were not only genotyped in breast cancer cases and controls who participated through blood sample donation, but were also genotyped in breast cancer cases who participated through tissue sample donation. None of the tagSNPs deviated significantly from HWE among the controls or showed a meaningful association with known breast cancer risk factors. Only one of the tagSNPs – TAG5 (also named I655V) in the *ERBB2 *gene – conferred an amino acid change in the protein product.

We estimated the risk of death from breast cancer associated with the tagSNPs (Table 2) in the *ATM*, *CHEK2 *and *ERBB2 *genes or their haplotypes (Table 3). We found a decreased risk of death from breast cancer associated with each addition of the rare TAG2 allele in the *CHEK2 *gene (*P *= 0.026) and an elevated risk of death from breast cancer associated with the rare TAG6 allele in the *CHEK2 *gene (*P *= 0.03), compared with homozygotes of the common allele for each variant. The associations did not, however, withstand Bonferroni correction. Carriers of haplotype 2 in the *CHEK2 *gene seemed to have a decreased risk of death from breast cancer (*P *= 0.038) compared with haplotype 1 carriers, whereas carriers of the rare *ERBB2 *haplotypes seemed to have an increased risk of death from breast cancer (*P *= 0.009). Neither association carried over to the global test (*P *= 0.15 and *P *= 0.45, respectively).

We noticed in Table 3 that all of the *ATM *haplotypes conferred a decrease in risk of death from breast cancer compared with haplotype 1. We therefore assessed the association of haplotype 1 with the risk of death from breast cancer and found a nonsignificantly elevated risk compared with noncarriers [odds ratio (OR), 1.13; 95% confidence interval (CI), 0.92–1.40].

### Tumour characteristics

We calculated global *P *values for the association between the tagSNP haplotypes in the *ATM*, *CHEK2 *and *ERBB2 *genes and the risk of tumour-characteristic-defined breast cancer from logistic regression models. Cases were divided into groups according to their tumour characteristics and each group contrasted against all controls. Each logistic regression model included the common haplotypes and the combined group of rare haplotypes, with the most common haplotype used as a reference standard. None of the global *P *values reached significance (supplementary Table 4), which indicates that none of the individual haplotypes affected the risk of developing tumours with certain characteristics.

We genotyped the *CHEK2 1100delC *gene mutation in our study population and have previously reported its effect on the overall risk of breast cancer [56]. The deletion was very rare in our study population, with a frequency of 0.7% among the cases and 0.4% among the controls. We could therefore not perform a meaningful analysis of the association between the deletion and breast cancer characteristics or survival in the current study.

### Overall risk of breast cancer: the *ATM *and *ERBB2 *genes

We found no effect of the *ATM *or *ERBB2 *tagSNPs (supplementary Table 5) or haplotypes (supplementary Table 6) on the overall risk of breast cancer, which was not altered after conditioning on the selection variables (menopausal hormone therapy and diabetes mellitus) or restricting the analyses to the randomly selected cases and controls. Stratifying the haplotype results by known breast cancer risk factors did not yield any additional compelling findings (supplementary Table 7).

We genotyped two mis-sense mutations in the *ATM *gene in the complete sample set: 4258 C→T (rs1800058; L1420F) and 2572 T→C (rs1800056; F858L). Neither mutation deviated significantly from HWE in controls. They were both rare in our study population, with a minor allele frequency of 1.9% for 4258 C→T and 1.4% for 2527 T→C in the controls. In exploring the change in the risk of breast cancer with each addition of the rare allele compared with noncarriers (assuming co-dominance), an elevated – but not significant – risk for the 4258 C→T (OR, 1.36; 95% CI, 0.91–2.04) was found, but no association emerged between the 2527 T→C and the risk of breast cancer (OR, 1.05; 95% CI, 0.65–1.71).

## Discussion

Although the gene products of *ATM, CHEK2 *and *ERBB2 *are involved in various aspects of breast cancer development and progression, our results suggest that common variation in these genes does not affect survival, tumour-characteristic-defined risk or the overall risk of breast cancer. We carefully studied these associations, both overall and in subgroups of known nongenetic breast cancer risk factors, using large population-based case–control material, and conclude that on the population level, common genetic variation in these genes is not of great importance for these outcomes. This does not preclude the possibility that more crucial – and rare – variation is influential in selected patient groups.

Our study was population-based. All participants were born in Sweden between 1919 and 1944, a time at which foreign immigration to Sweden was still rare [57], which means population stratification is of limited concern in our study. To minimize exposure misclassification, we applied genotyping methods with low error rates (the Sequenom and Illumina methods have genotyping error rates of 0.5% and 0.3%, respectively), DNA samples were randomly assigned to the genotyping plates and the genotyping personnel were blinded to case–control status. Furthermore, we replicated genotype calls of 200 randomly selected SNPs for a subset of samples using a separate genotyping method with >99.5% concordance.

The oestrogen and progesterone receptor status of tumours and S-phase fraction were assessed at seven different laboratories in Sweden, but it is doubtful that the genotype frequencies could be related to any interlaboratory differences. A large proportion of the information on receptor status, S-phase fraction and grade was missing. Assessment of receptor status and S-phase fraction was, to a large extent, dependent on the size of the tumour, but evaluation of the tumour grade was mostly dependent on the pathologist's decision. Because genotype frequencies were not related to tumour size, bias owing to the missing information on these factors seems unlikely.

Survival bias could be a concern in our study because nonparticipation was related to severe disease or death. However, we obtained the majority of the tissue samples requested for deceased patients with breast cancer or breast cancer cases who had declined donation of a blood sample. The lack of tissue accessibility is unlikely to be related to *ATM*, *ERBB2 *or *CHEK2 *genetic variation because it depended on the inability of the respective pathology department to retrieve the samples. The genotype frequencies of the tagSNPs in the *ERBB2 *and *CHEK2 *genes did not differ between blood and tissue samples, suggesting that the survival bias was negligible. We were not able to genotype TAG1, TAG2, TAG3, TAG6 and TAG7 in the *ATM *gene in the tissue samples, but because the results were not different in the analyses restricted to the most severe cases among those who donated blood samples indicates that this was not a major problem in our study.

The loss of power from testing SNPs indirectly is thought to be related to the *R*^2 ^measure [46,58], but exceptions to these assumptions have been reported [59]. We assessed the capability of the tagSNPs to convey an association signal from unobserved, in addition to observed, SNPs. We captured the unobserved SNPs with average *R*^2 ^values of 0.92, 0.72 and 0.93 in the *ATM*, *ERBB2 *and *CHEK2*, genes, respectively, and thus suffered minimal loss of power owing to indirect testing. We performed standard power calculations assuming α = 0.05, thus giving an indication regarding the general power of our study. We acknowledge, however, that the power is reduced in the subgroup analyses (an effect cannot be excluded in the smallest groups) and at lower α-levels (required when multiple tests are carried out). For the ability of haplotypes to predict the allele count at a causal locus with a minor allele frequency of 0.20, we had 89% power for *ATM*, 73% power for *ERBB2 *and 87% power for *CHEK2 *to detect an odds ratio of 1.3 in the risk component of the study. To detect a hazard ratio of 1.4, with an α-level of 0.05, in the survival component of the study, we had 50% power for TAG1 in *CHEK2*, which had a minor allele frequency of 0.13, and 76% power for TAG5 in *CHEK2*, which had a minor allele frequency of 0.38.

Our data did not support an association between common variation in the *ATM*, *CHEK2 *and *ERBB2 *genes and breast cancer survival or the risk of developing tumours of different characteristics. Hence, we did not confirm the finding of Han and colleagues who found that an *ERBB2 *haplotype composed of two nonsynonymous tagSNPs – I655V and P1170A – increased the risk of breast cancer death or recurrence [35]. We included the I655V as a tagSNP in our study and genotyped the P1170A in the 92 controls. We found no effect of the I655V on breast cancer survival or tumour characteristics defined breast cancer.

To our knowledge, no study has investigated *ATM *or *CHEK2 *common haplotypes in relation to breast cancer survival or tumour characteristics, although one study explored the effect of three common polymorphisms in *ATM *and two polymorphisms in *CHEK2 *on breast cancer survival [60]. This study found no association, which is in agreement with our findings. The rare *1100delC *mutation in the *CHEK2 *gene has been associated with breast tumours of high grade [61], in addition to steroid receptor-positive breast tumours, but not with overall survival [62]. The mutation was too rare in our study population to be investigated in relation to breast cancer survival or tumour characteristics.

We previously found no association between common variation in the *CHEK2 *gene and overall risk of breast cancer [56], which was in agreement with earlier findings [63]. In this study, we, correspondingly, found no effect of common variation in the *ATM *or *ERBB2 *genes on the risk of breast cancer, even when the results were stratified by known breast cancer risk factors. One study of the *ATM *gene [64] and two studies of the *ERBB2 *gene [35,65] are in agreement with our findings. Tamimi and colleagues found no association between the haplotypes of five Hapmap tagSNPs (one of which was TAG7 in our study) in the *ATM *gene and the risk of breast cancer [64]. Benusiglio and colleagues explored *ERBB2 *haplotypes composed of five tagSNPs [three of which were TAG2, TAG3 and TAG5 in our study] – including the nonsynonymous I655V and P1170A – in relation to the risk of breast cancer [65], whereas Han and colleagues solely studied the I655V and P1170A as tagSNPs [35]. Neither study found any effect of the haplotypes on the risk of breast cancer. Common haplotypes in the *ERBB2 *gene thus do not seem to affect the risk of breast cancer, although results regarding the I655V common variant in the *ERBB2 *gene have been conflicting [35,65-67]. We found no association of the I655V with the risk of breast cancer.

Three groups have found an association between specific *ATM *common haplotypes and the risk of breast cancer [68-70]. Lee and colleagues [70] and Koren and colleagues [68] reconstructed haplotypes in the *ATM *gene from five and eight randomly selected common SNPs, respectively, whereas Angele and colleagues [69] included 11 common SNPs in the *ATM *gene for their haplotype estimation that had been either previously reported in the literature or detected by sequencing. SNP selection overlapped somewhat between the three studies, but none of them reported the probability of their SNPs being able to predict underlying variation in the gene. Furthermore, findings from two of the three groups [68,69] were derived from small sample sizes.

The 4258 C→T and 2527 T→C mutations in the *ATM *gene have been detected in breast cancer patients [69,71-73], but to the best of our knowledge, these mutations have not been reported in A-T patients. In line with two [69,72] out of three reports [69,71,72], we found an elevated – but not significant – risk of breast cancer in carriers of the rare 4258 T allele in the *ATM *gene. We did not, however, find such an association in carriers of the 2527 C allele. One study found a twofold increase in the risk of breast cancer that was related to the 2527 T→C mutation in a population from USA, but did not confirm the finding in a Polish population [73]. Three other groups did not detect any significant effect of the 2527 T→C mutation on the risk of breast cancer [69,71,72], although one of the groups found elevated point estimates [72].

Although the 4258 C→T and 2527 T→C mis-sense variants do not seem to target residues known to be crucial for the function of the ATM protein [72], an increasing amount of evidence suggests that mis-sense variants in the *ATM *gene cause chromosomal instability and abolish the radiation-induced kinase activity of ATM [74]. Mutant ATM protein also seems to interfere with normal ATM function in a dominant-negative manner [74]. Previous mutation screening studies have indicated that mis-sense mutations in the *ATM *gene – rather than protein-truncating mutations – are over-represented in patients with breast cancer compared with the general population [72,75-79]. A recent publication refuted this and found that *ATM *gene mutations that cause A-T – that is, truncating, splicing and mis-sense mutations – are breast cancer susceptibility alleles [80]. They found a greater than twofold increase in the risk of breast cancer related to a combination of 12 mutations, including six truncating mutations [80]. Thus, controversy remains both regarding which type of mutations in the *ATM *gene are involved in breast cancer aetiology and which mutations actually drive the association with the risk of breast cancer [69-73,81-85].

## Conclusion

Our study had a sound epidemiological design and a comprehensive coverage of SNPs. The results indicate that common variation in the *ATM*, *CHEK2 *or *ERBB2 *genes does not have a role in breast cancer aetiology or progression.

## Abbreviations

A-T = ataxia-telangiectasia; ATM = ataxia-telangiectasia mutated; CHEK2 = checkpoint kinase 2; CI = confidence interval; dbSNP = Single Nucleotide Polymorphism database; ERBB2 = v-erb-b2 avian erythroblastic leukemia viral oncogene homolog 2; HWE = Hardy–Weinberg equilibrium; kb = kilobase; LD = linkage disequilibrium; OR = odds ratio; SNP = single nucleotide polymorphism; tagSNP = haplotype-tagging SNP.

## Competing interests

The authors declare that they have no competing interests.

## Authors' contributions

KE wrote the article and performed the statistical analyses with the assistance of KH and YL. LUR and SW organized data and sample collection. CB oversaw the genotyping procedures and KE assisted on the genotyping. KE and YL selected the polymorphisms. SW and JL co-ordinated the study. KH, JP, KSC, ETL, PH, JL and SW contributed to the conception and design of the project and crucially revised the manuscript. All authors read and approved the final manuscript.

## Supplementary Material

Additional file 1 Microsoft Word document containing supplementary figures 1 and 2, and supplementary tables 1 to 7. These detail the statistics for the genotyping results, successfully genotyped SNPs in the *ATM *and *ERBB2 *genes, risk of breast cancer associated with the *ATM *and *ERBB2 *genes, association of the *ATM*, *CHEK2 *and *ERBB2 *genes with the risk of tumour-characteristic-defined breast cancer and LD plots of the *ATM *and *ERBB2 *genes. Click here for file

## Figures and Tables

**Table 1:** Characteristics of the breast cancer cases in the parent study and the present genetic study

	Parent study	Present study	Present study	
			
Characteristic	*n *(%)	*n *(%)	Breast cancer deaths^a^	Mortality rate^b^
			
Total cases	2,817	1,579	198	0.016
Tumour size (mm)				
≤20	1,858 (71.4)	1,087 (73.6)	100	0.010
21–30	489 (18.8)	270 (18.3)	57	0.026
>30	254 (9.8)	120 (8.1)	38	0.043
Unknown	216	102	3	0.022
*P *value^c^		0.13		
TNM stage				
1	1,392 (54.4)	804 (55.3)	40	0.006
2	1,074 (42.0)	606 (41.7)	126	0.026
3	58 (2.3)	31 (2.1)	15	0.076
4	34 (1.3)	12 (0.8)	11	0.262
Unknown	259	126	6	0.019
*P *value^c^		0.39		
Lymph-node involvement				
No	1,741 (68.2)	1,002 (68.9)	68	0.008
Yes	813 (31.8)	453 (31.1)	122	0.035
Unknown	263	124	8	0.027
*P *value^c^		0.23		
Grade				
1	259 (14.7)	143 (14.2)	4	0.003
2	739 (41.9)	444 (44.1)	37	0.010
3	766 (43.4)	419 (41.7)	93	0.027
Unknown	1,053	573	64	0.016
*P *value^c^		0.61		
Oestrogen and progesterone receptor status				
ER^+^/PR^+^	1,162 (62.7)	677 (65.4)	72	0.012
ER^+^/PR-	285 (15.4)	133 (12.9)	28	0.026
ER-/PR^+^	69 (3.7)	33 (3.2)	7	0.027
ER-/PR-	338 (18.2)	192 (18.6)	52	0.037
Unknown	963	544	39	0.010
*P *value^c^		0.37		
Histological type				
Ductal	1,891 (72.5)	1,070 (72.4)	147	0.016
Lobular	304 (11.7)	185 (12.5)	19	0.012
Other	414 (15.9)	223 (15.1)	28	0.014
Unknown	208	101	4	0.039
*P *value^c^		0.48		
S-phase fraction				
Low	689 (68.1)	401 (70.0)	40	0.011
High	323 (31.9)	172 (30.0)	52	0.040
Unknown	1805	1,006	106	0.013
*P *value^c^		0.71		

^a^From the date of diagnosis until 31 December 2003.

^b^From the date of diagnosis until 31 December 2003 or until the date of death or emigration, whichever came first. Calculated as breast cancer deaths per person-year of follow-up.

^c^Chi-squared test of independence between the parent and the present studies.

ER, oestrogen receptor; PR, progesterone receptor

**Table 2:** Characteristics of the *ATM*, *CHEK2 *and *ERBB2 *tagSNPs and their association with breast cancer survival

SNP ID	SNP name	Number of cases/controls	Minor allele frequency^a^	Breast cancer deaths/person-years^b^	HR (95% CI)^b,c^
		
*ATM*					
TAG1^d^	rs4987886	1,220/1,440	0.06	70/10,660	0.86 (0.42–1.76)
TAG2^d^	rs3092991	1,119/1,318	0.14	65/9,717	1.12 (0.71–1.77)
TAG3^d^	rs1800057	1,144/1,346	0.03	66/9,958	0.62 (0.16–2.46)
TAG4	rs1801516	1538/1,500	0.15	185/12,421	0.99 (0.75–1.30)
TAG5	rs17107917	1,546/1,493	0.04	192/12,466	1.01 (0.63–1.64)
TAG6^d^	rs227060	1,152/1,350	0.28	68/10,004	0.77 (0.52–1.14)
TAG7^d^	rs664143	1,227/1,408	0.48	73/10,684	1.24 (0.89–1.73)
		
*CHEK2*					
TAG1	rs8135424	1,539/1,478	0.13	197/12,367	1.08 (0.83–1.41)
TAG2	rs5762749	1,516/1,472	0.35	191/12,172	0.78 (0.62–0.97)
TAG3	rs743185	1,547/1,491	0.12	192/12,471	1.21 (0.91–1.62)
TAG4	rs738722	1,501/1,444	0.25	187/12,083	0.72 (0.56–0.93)
TAG5	rs5762765	1,510/1,456	0.38	192/12,118	1.15 (0.94–1.41)
TAG6	rs2236142	1,541/1,471	0.31	192/12,398	1.25 (1.02–1.54)
		
*ERBB2*					
TAG1	rs2643195	1,494/1,458	0.32	186/12,052	1.09 (0.88–1.35)
TAG2	rs4252596	1,530/1,481	0.13	189/12,330	0.95 (0.70–1.30)
TAG3	rs2952155	1,459/1,407	0.25	184/11,666	1.02 (0.80–1.29)
TAG4	rs2952156	1,546/1,481	0.32	194/12,445	1.11 (0.89–1.37)
TAG5^e^	rs1801200^e^	1,548/1,485	0.26	193/12,463	1.00 (0.80–1.25)
TAG6	rs4252665	1,527/1,486	0.05	182/12,401	0.76 (0.45–1.27)
TAG7	rs3809717	1,532/1,478	0.31	194/12,310	0.96 (0.77–1.20)

^a^In controls.

^b^Among women with breast cancer.

^c^HRs are assessed assuming co-dominance and show the increase/decrease in risk of death from breast cancer with each addition of the minor allele compared with homozygotes of the major allele.

^d^Not genotyped in the patients who participated through tissue sample donation.

^e^Also named I655V.

ATM, ataxia-telangiectasia mutated; CHEK2, checkpoint kinase 2; CI, confidence interval; ERBB2, v-erb-b2 avian erythroblastic leukemia viral oncogene homolog 2; HR, hazard ratio; SNP, single nucleotide polymorphism; tagSNP, haplotype-tagging SNP.

**Table 3:** tagSNP haplotypes in the *ATM*, *CHEK2 *and *ERBB2 *genes in relation to breast cancer survival

Haplotype no.	Haplotypes	Haplotype proportions (cases)	HR (95% CI)
*ATM*		*n *= 1,574^a^	
1	AACGCCT	0.414	1.00 (Reference)
2	AACGCTC	0.231	0.85 (0.65–1.13)
3	AGCACCC	0.150	0.89 (0.66–1.21)
4	AACGCCC	0.062	0.86 (0.52–1.44)
5	TACGCCT	0.064	0.88 (0.55–1.40)
6	AACGGTC	0.043	0.95 (0.57–1.57)
	Rare^b^	0.037	0.95 (0.53–1.68)
Global *P *value^c^			0.95

*CHEK2*		*n *= 1,571^a^	
1	GCCCCC	0.223	1.00 (Reference)
2	GGCTGC	0.231	0.72 (0.52–0.98)
3	GCCCCG	0.140	1.02 (0.72–1.43)
4	ACCCGC	0.113	1.00 (0.70–1.41)
5	GCTCGG	0.089	1.08 (0.73–1.57)
6	GGCCGC	0.052	0.61 (0.34–1.10)
7	GCCCGC	0.027	0.60 (0.26–1.41)
	Rare^d^	0.125	0.95 (0.67–1.35)
Global *P *value^c^			0.15

*ERBB2*		*n *= 1,579^a^	
1	GGCGACT	0.296	1.00 (Reference)
2	AGTAACG	0.166	0.98 (0.71–1.34)
3	GGCGGCG	0.135	1.01 (0.73–1.40)
4	GACGACG	0.116	0.98 (0.69–1.39)
5	AGTAGCG	0.075	1.21 (0.81–1.81)
6	AGCAACG	0.068	1.16 (0.76–1.77)
7	GGCGACG	0.079	1.06 (0.69–1.63)
8	GGCGGTG	0.048	0.81 (0.47–1.39)
	Rare^e^	0.018	2.21 (1.22–4.02)
Global *P *value^c^			0.45

^a^Information on at least one tagSNP.

^b^11 rare haplotypes combined. Each haplotype has frequency <3% among the controls.

^c^Probability ratio test.

^d^19 rare haplotypes combined. Each haplotype has frequency <3% among the controls.

^e^19 rare haplotypes combined. Each haplotype has frequency <3% among the controls.

ATM, ataxia-telangiectasia mutated; CHEK2, checkpoint kinase 2; CI, confidence interval; ERBB2, v-erb-b2 avian erythroblastic leukemia viral oncogene homolog 2; HR, hazard ratio; tagSNP, haplotype-tagging SNP.
