# Supplementary material for: Comprehensive analysis of the ATM, CHEK2 and ERBB2 genes in relation to breast tumour characteristics and survival: a population-based case-control and follow-up study
Source: Breast Cancer Res. 2006 Nov 28;8(6):R67. doi: 10.1186/bcr1623 (PMC1797028; doi:10.1186/bcr1623)
Supplement: Additional file 1 — Microsoft Word document containing supplementary figures 1 and 2, and supplementary tables 1 to 7. These detail the statistics for the genotyping results, successfully genotyped SNPs in the ATM and ERBB2 genes, risk of breast cancer associated with the ATM and ERBB2 genes, association of the ATM, CHEK2 and ERBB2 genes with the risk of tumour-characteristic-defined breast cancer and LD plots of the ATM and ERBB2 genes. [file bcr1623-S1.doc]

Supplementary Table 1. Summary statistics on genotyping results and SNP coverage in *ATM*, *CHEK2* and *ERBB2*.

| Summary statistics | *ATM* | *CHEK2* | *ERBB2* |
| --- | --- | --- | --- |
| # successfully genotyped SNPs^a^ | 152^b^ | 34^c^ | 38^d^ |
| # polymorphic SNPs | 68 | 23 | 16 |
| # common SNPs^e^ | 52 | 19 | 13 |
| # SNPs deviating from HWE^f^ | 1 | 5 | 0 |
|  |  |  |  |
| # SNPs included in study | 51 | 14 | 13 |
|  |  |  |  |
| Sequence coverage (kb) | 146.2 | 52.0 | 33.9 |
| Mean spacing between SNPs (kb) | 2.9 | 4.0 | 2.8 |
| Median spacing between SNPs (kb) | 2.0 | 3.2 | 2.7 |
| # common haplotypes^e,g^ | 6 | 6 | 8 |
| Chromosomes accounted for by common haplotypes (%)^g^ | 89 | 81 | 96 |
| # tagSNPs selected | 7 | 6 | 7 |
| Average tagSNP prediction of common SNPs included in study (*R^2^*)^e^ | 0.96 | 0.95 | 0.99 |
| Average tagSNP prediction of common haplotypes (*R^2^*)^e^ | 0.95 | 0.94 | 0.94 |
|  |  |  |  |
| Coverage evaluation^h^ |  |  |  |
| Average prediction of dropped SNPs (*R^2^*) | 0.92 | 0.93 | 0.72 |
| Percentage of *R^2^* values ≥ 0.7 | 92 | 93 | 70 |

^a^ In 92 controls.

^b^ Supplementary Table 2.

^c^ Table 2 in Einarsdóttir et al. [36].

^d^ Supplementary Table 3.

^e^ Common was defined as minor allele frequency ≥ 0.03 (SNPs) or haplotype frequency ≥ 0.03.

^f^ *P* < 0.01

^g^ Haplotypes reconstructed from all SNPs genotyped in 92 controls.

^h^ SNP dropping method by Weale et al. [68].

Supplementary Table 2. 152 SNPs in *ATM* and its 10kb flanking sequences successfully genotyped in 92 controls.

| SNP no. | dbSNP name | Position^a^ | Alleles^b^ | Minor allele  frequency^c^ | HWE  P-value^d^ | tagSNPs |
| --- | --- | --- | --- | --- | --- | --- |
| 1 | rs1442729 | 107596387 | C | 0 | --- |  |
| 2 | rs4987874 | 107597513 | C/G | 0.02 | 0.83 |  |
| 3 | rs189037 | 107599043 | A/G | 0.39 | 0.04 |  |
| 4 | rs3092854 | 107600456 | C | 0 | --- |  |
| 5 | rs4987886 | 107601263 | A/T | 0.08 | 0.61 | TAG1 |
| 6 | rs228591 | 107602543 | G/A | 0.44 | 0.65 |  |
| 7 | rs4987897 | 107603352 | C | 0 | --- |  |
| 8 | rs3218693 | 107603453 | A | 0 | --- |  |
| 9 | rs7112053 | 107603568 | C | 0 | --- |  |
| 10 | rs4987899 | 107604071 | T | 0 | --- |  |
| 11 | rs641605 | 107607129 | A/G | 0.41 | 0.35 |  |
| 12 | rs11212562 | 107609908 | T/C | 0.01 | 0.96 |  |
| 13 | rs4987908 | 107610295 | A | 0 | --- |  |
| 14 | rs2234996 | 107611536 | A/G | 0.04 | 0.70 |  |
| 15 | rs1442730 | 107611616 | G | 0 | --- |  |
| 16 | rs2234997 | 107611653 | T | 0 | --- |  |
| 17 | rs3218710 | 107611845 | G | 0 | --- |  |
| 18 | rs623860 | 107611992 | T/C | 0.39 | 0.34 |  |
| 19 | rs228598 | 107612707 | G/T | 0.39 | 0.12 |  |
| 20 | rs7113958 | 107612787 | T | 0 | --- |  |
| 21 | rs228599 | 107612870 | A/G | 0.38 | 0.37 |  |
| 22 | rs12287161 | 107613134 | G | 0 | --- |  |
| 23 | rs4753834 | 107618188 | G/T | 0.23 | 0.48 |  |
| 24 | rs228588 | 107618541 | C/G | 0.41 | 0.50 |  |
| 25 | rs3218707 | 107619937 | G | 0 | --- |  |
| 26 | rs2235004 | 107620093 | T | 0 | --- |  |
| 27 | rs3218706 | 107620749 | A | 0 | --- |  |
| 28 | rs677068 | 107621323 | T/C | 0.01 | 0.96 |  |
| 29 | rs4987923 | 107621622 | C/G | 0.02 | 0.83 |  |
| 30 | rs4987924 | 107621692 | A/C | 0.02 | 0.83 |  |
| 31 | rs600931 | 107622545 | A/G | 0.41 | 0.41 |  |
| 32 | rs694376 | 107624258 | T/G | 0.41 | 0.57 |  |
| 33 | rs1800727 | 107624980 | C | 0 | --- |  |
| 34 | rs4987945 | 107627802 | C | 0 | --- |  |
| 35 | rs228592 | 107628399 | A/C | 0.41 | 0.41 |  |
| 36 | rs4987948 | 107629267 | C | 0 | --- |  |
| 37 | rs4987953 | 107630279 | A | 0 | --- |  |
| 38 | rs2229019 | 107632220 | C | 0 | --- |  |
| 39 | rs11212568 | 107633256 | T | 0 | --- |  |
| 40 | rs2235011 | 107633448 | A | 0 | --- |  |
| 41 | rs4987963 | 107634705 | T | 0 | --- |  |
| 42 | rs672655 | 107634867 | G/A | 0.41 | 0.41 |  |
| 43 | rs627418 | 107636435 | G/A | 0.41 | 0.57 |  |
| 44 | rs1800056 | 107643213 | T/C | 0.02 | 0.83 |  |
| 45 | rs3218673 | 107643255 | C | 0 | --- |  |
| 46 | rs2234994 | 107644330 | G | 0 | --- |  |
| 47 | rs3218687 | 107644393 | A | 0 | --- |  |
| 48 | rs3218708 | 107644512 | C | 0 | --- |  |
| 49 | rs4987972 | 107645057 | C | 0 | --- |  |
| 50 | rs3092991 | 107645726 | A/G | 0.12 | 0.05 | TAG2 |
| 51 | rs4987975 | 107645819 | C | 0 | --- |  |
| 52 | rs637064 | 107646119 | T/C | 0.40 | 0.23 |  |
| 53 | rs1064815 | 107647274 | A | 0 | --- |  |
| 54 | rs3218679 | 107647404 | G | 0 | --- |  |
| 55 | rs2301194 | 107647545 | C | 0 | --- |  |
| 56 | rs3092857 | 107648509 | A | 0 | --- |  |
| 57 | rs3092858 | 107648529 | T | 0 | --- |  |
| 58 | rs3092859 | 107648541 | T | 0 | --- |  |
| 59 | rs1800057 | 107648666 | C/G | 0.03 | 0.75 | TAG3 |
| 60 | rs618499 | 107654049 | G/A | 0.37 | 0.39 |  |
| 61 | rs3092851 | 107657133 | A | 0 | --- |  |
| 62 | rs1003623 | 107657792 | T/C | 0.40 | 0.27 |  |
| 63 | rs624366 | 107659307 | C/G | 0.39 | 0.20 |  |
| 64 | rs3092842 | 107660415 | G | 0 | --- |  |
| 65 | rs7121757 | 107660520 | G | 0 | --- |  |
| 66 | rs654005 | 107660607 | A/G | 0.40 | 0.30 |  |
| 67 | rs668208 | 107661513 | A | 0 | --- |  |
| 68 | rs592955 | 107661683 | A/C | 0.41 | 0.46 |  |
| 69 | rs609261 | 107663344 | G/A | 0.40 | 0.36 |  |
| 70 | rs11212580 | 107664374 | G | 0 | --- |  |
| 71 | rs3092856 | 107664942 | C | 0 | --- |  |
| 72 | rs1800058 | 107665560 | C/T | 0.01 | 0.91 |  |
| 73 | rs1800889 | 107668697 | C | 0 | --- |  |
| 74 | rs600329 | 107668943 | C/T | 0.41 | 0.41 |  |
| 75 | rs3218678 | 107670844 | A | 0 | --- |  |
| 76 | rs681479 | 107670914 | A | 0 | --- |  |
| 77 | rs681518 | 107670946 | A | 0 | --- |  |
| 78 | rs645485 | 107674073 | G/A | 0.40 | 0.25 |  |
| 79 | rs684542 | 107675198 | T | 0 | --- |  |
| 80 | rs4988031 | 107676672 | T | 0 | --- |  |
| 81 | rs4988034 | 107677135 | T | 0 | --- |  |
| 82 | rs4988037 | 107678208 | C/T | 0.04 | 0.70 |  |
| 83 | rs3092909 | 107678728 | G | 0 | --- |  |
| 84 | rs4988040 | 107679260 | T | 0 | --- |  |
| 85 | rs650128 | 107679401 | T/C | 0.41 | 0.41 |  |
| 86 | rs4988042 | 107680038 | A | 0 | --- |  |
| 87 | rs3092829 | 107680604 | T/C | 0.02 | 0.87 |  |
| 88 | rs1801516 | 107680672 | G/A | 0.13 | 0.19 | TAG4 |
| 89 | rs1801673 | 107680673 | A | 0 | --- |  |
| 90 | rs599558 | 107682748 | A/G | 0.41 | 0.41 |  |
| 91 | rs3218686 | 107683948 | G/A | 0.01 | 0.96 |  |
| 92 | rs4988059 | 107684040 | G | 0 | --- |  |
| 93 | rs2301195 | 107684355 | G | 0 | --- |  |
| 94 | rs3092911 | 107686268 | A | 0 | --- |  |
| 95 | rs660429 | 107686721 | C/A | 0.41 | 0.41 |  |
| 96 | rs673281 | 107687279 | A/G | 0.37 | 0.01 |  |
| 97 | rs676004 | 107687887 | T | 0 | --- |  |
| 98 | rs659243 | 107688377 | G | 0 | --- |  |
| 99 | rs4988077 | 107689641 | C | 0 | --- |  |
| 100 | rs4988079 | 107689735 | A | 0 | --- |  |
| 101 | rs4988081 | 107690413 | T | 0 | --- |  |
| 102 | rs634268 | 107691468 | A/G | 0.01 | 0.91 |  |
| 103 | rs11212587 | 107691820 | G | 0 | --- |  |
| 104 | rs3092826 | 107691960 | T | 0 | --- |  |
| 105 | rs4988084 | 107692166 | T | 0 | --- |  |
| 106 | rs17107917 | 107693249 | C/G | 0.06 | 0.22 | TAG5 |
| 107 | rs7942747 | 107693785 | C | 0 | --- |  |
| 108 | rs4988089 | 107695274 | A | 0 | --- |  |
| 109 | rs3218677 | 107697369 | G | 0 | --- |  |
| 110 | rs676570 | 107697848 | A | 0 | --- |  |
| 111 | rs11212588 | 107698778 | A/G | 0.01 | 0.91 |  |
| 112 | rs595747 | 107699283 | T/C | 0.41 | 0.57 |  |
| 113 | rs662578 | 107699767 | T/C | 0.41 | 0.41 |  |
| 114 | rs4988101 | 107700733 | C | 0 | --- |  |
| 115 | rs3092992 | 107700989 | A/C | 0.03 | 0.79 |  |
| 116 | rs609429 | 107701719 | G/C | 0.41 | 0.41 |  |
| 117 | rs1800061 | 107702047 | G | 0 | --- |  |
| 118 | rs3092831 | 107703610 | C | 0 | --- |  |
| 119 | rs1060793 | 107706301 | A | 0 | --- |  |
| 120 | rs12792858 | 107707022 | A | 0 | --- |  |
| 121 | rs4988127 | 107709173 | G | 0 | --- |  |
| 122 | rs609655 | 107709463 | T/C | 0.41 | 0.57 |  |
| 123 | rs7106001 | 107709635 | A | 0 | --- |  |
| 124 | rs3218704 | 107709728 | C | 0 | --- |  |
| 125 | rs227060 | 107710091 | C/T | 0.38 | 0.03 | TAG6 |
| 126 | rs4988129 | 107710157 | DEL/T | 0.40 | 0.27 |  |
| 127 | rs4988132 | 107710428 | T | 0 | --- |  |
| 128 | rs227061 | 107710539 | G/A | 0.39 | 0.15 |  |
| 129 | rs227062 | 107710593 | A/G | 0.43 | 0.56 |  |
| 130 | rs227064 | 107712603 | G/A | 0.41 | 0.57 |  |
| 131 | rs227065 | 107713149 | G/A | 0.01 | 0.96 |  |
| 132 | rs11212590 | 107713205 | T/G | 0.01 | 0.96 |  |
| 133 | rs7927843 | 107714618 | G | 0 | --- |  |
| 134 | rs12278194 | 107718890 | T | 0 | --- |  |
| 135 | rs3017873 | 107719337 | T | 0 | --- |  |
| 136 | rs227074 | 107720305 | G/A | 0.40 | 0.23 |  |
| 137 | rs364613 | 107722600 | G | 0 | --- |  |
| 138 | rs1657973 | 107722838 | A | 0 | --- |  |
| 139 | rs227075 | 107723406 | C/T | 0.39 | 0.34 |  |
| 140 | rs425538 | 107724549 | A/C | 0.41 | 0.57 |  |
| 141 | rs11825355 | 107724851 | A | 0 | --- |  |
| 142 | rs17412927 | 107726231 | G/C | 0.04 | 0.70 |  |
| 143 | rs4986839 | 107729825 | A/C | 0.04 | 0.70 |  |
| 144 | rs664143 | 107730871 | C/T | 0.42 | 0.66 | TAG7 |
| 145 | rs652541 | 107731235 | C/T | 0.39 | 0.34 |  |
| 146 | rs227053 | 107732065 | A/T | 0.39 | 0.34 |  |
| 147 | rs2283264 | 107732779 | C | 0 | --- |  |
| 148 | rs227094 | 107739010 | A/C | 0.40 | 0.30 |  |
| 149 | rs170548 | 107740046 | T/G | 0.32 | 0.01 |  |
| 150 | rs12284748 | 107742220 | G | 0 | --- |  |
| 151 | rs652311 | 107745279 | A/G | 0.40 | 0.09 |  |
| 152 | rs11212595 | 107745580 | G/A | 0.01 | 0.96 |  |

^a^ dbSNP build 125

^b^ Major alleles given first and minor alleles second

^c^ In 92 controls

^d^ From a χ^2^ test in 92 controls

Supplementary Table 3. 38 SNPs in *ERBB2* and its 10kb flanking sequences successfully genotyped in 92 controls.

| SNP no. | dbSNP name | Position^a^ | Alleles^b^ | Minor allele  frequency^c^ | HWE  P-value^d^ | tagSNPs |
| --- | --- | --- | --- | --- | --- | --- |
| 1 | rs8182267 | 35104837 | G/A | 0.01 | 0.96 |  |
| 2 | rs2643195 | 35106644 | G/A | 0.31 | 0.46 | TAG1 |
| 3 | rs4252596 | 35109360 | C/A | 0.11 | 0.89 | TAG2 |
| 4 | rs4252599 | 35109644 | C | 0 | --- |  |
| 5 | rs4252600 | 35110508 | G | 0 | --- |  |
| 6 | rs7216731 | 35110643 | C | 0 | --- |  |
| 7 | rs9904609 | 35110714 | G | 0 | --- |  |
| 8 | rs1565923 | 35112204 | G/A | 0.30 | 0.53 |  |
| 9 | rs2952155 | 35115244 | C/T | 0.23 | 0.15 | TAG3 |
| 10 | rs4252604 | 35115627 | C | 0 | --- |  |
| 11 | rs4252605 | 35115703 | C | 0 | --- |  |
| 12 | rs4252608 | 35116492 | C/T | 0.02 | 0.87 |  |
| 13 | rs4252613 | 35118255 | C | 0 | --- |  |
| 14 | rs1810132 | 35119531 | T/C | 0.32 | 0.68 |  |
| 15 | rs4252625 | 35121874 | C | 0 | --- |  |
| 16 | rs4252627 | 35122241 | T/C | 0.33 | 0.68 |  |
| 17 | rs4252628 | 35122440 | T | 0 | --- |  |
| 18 | rs2934967 | 35123904 | A/G | 0.32 | 0.71 |  |
| 19 | rs4252632 | 35125531 | G | 0 | --- |  |
| 20 | rs4252634 | 35125576 | A | 0 | --- |  |
| 21 | rs2934968 | 35126479 | A | 0 | --- |  |
| 22 | rs4252636 | 35126656 | G | 0 | --- |  |
| 23 | rs4252639 | 35129705 | C/G | 0.02 | 0.87 |  |
| 24 | rs4252643 | 35130293 | A | 0 | --- |  |
| 25 | rs2952156 | 35130361 | G/A | 0.31 | 0.31 | TAG4 |
| 26 | rs1801200^e^ | 35133114 | A/G | 0.26 | 0.58 | TAG5^e^ |
| 27 | rs903506 | 35133288 | A/G | 0.33 | 0.68 |  |
| 28 | rs4252645 | 35133835 | A | 0 | --- |  |
| 29 | rs4252648 | 35134420 | G | 0 | --- |  |
| 30 | rs4252655 | 35136701 | C | 0 | --- |  |
| 31 | rs1058808^f^ | 35137563 | G/C | 0.34 | 0.56 |  |
| 32 | rs2230700 | 35137828 | G | 0 | --- |  |
| 33 | rs4252661 | 35138414 | T | 0 | --- |  |
| 34 | rs4252664 | 35138827 | C | 0 | --- |  |
| 35 | rs4252665 | 35138909 | C/T | 0.04 | 0.70 | TAG6 |
| 36 | rs3809717 | 35140512 | G/T | 0.32 | 0.14 | TAG7 |
| 37 | rs8066837 | 35141205 | C | 0 | --- |  |
| 38 | rs4794818 | 35147259 | C | 0 | --- |  |

^a^ dbSNP build 125

^b^ Major alleles given first and minor alleles second

^c^ In 92 controls

^d^ From a χ^2^ test in 92 controls

^e^ Also named I655V

^f^ Also named P1170A


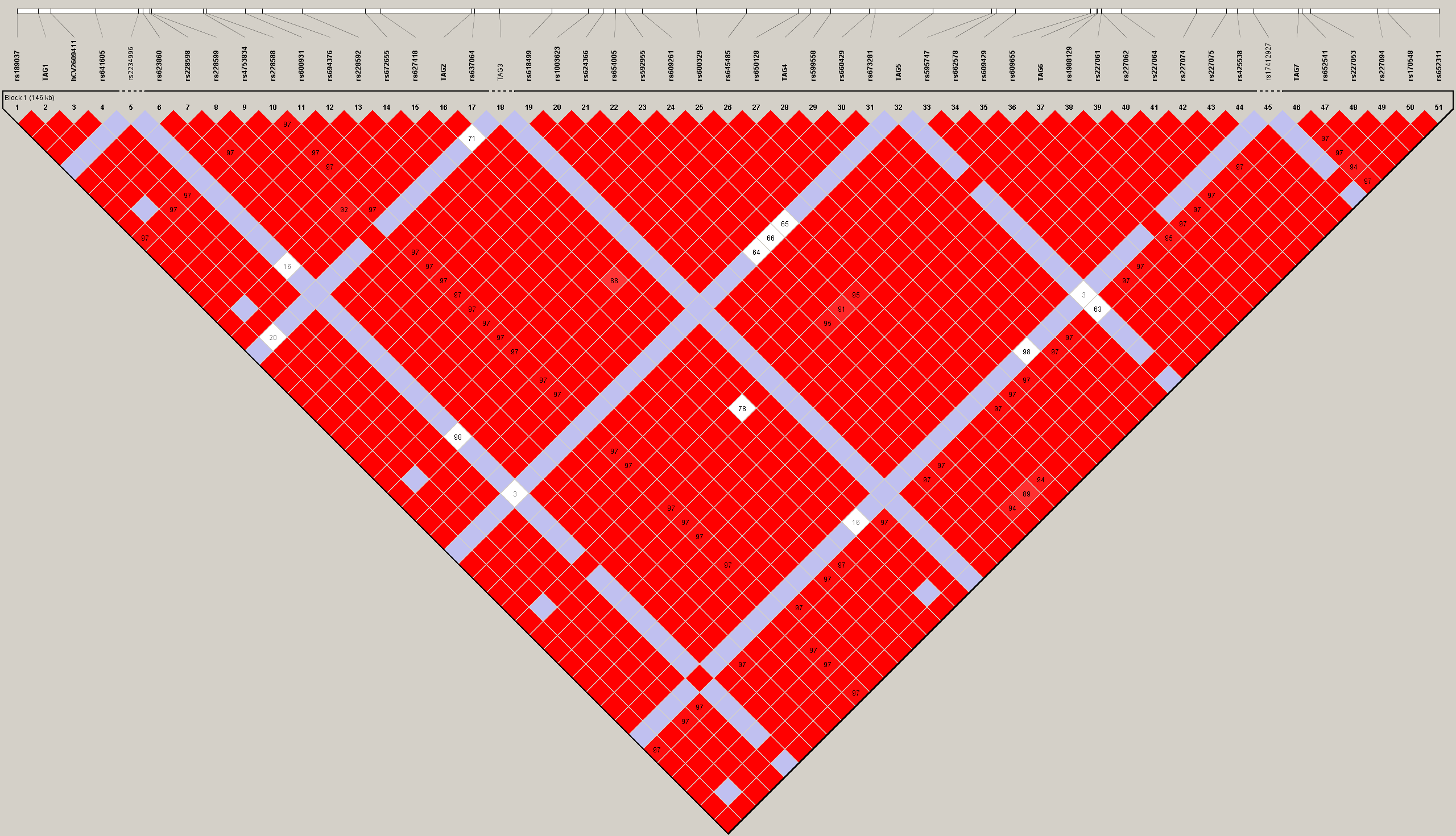


Supplementary Figure 1. LD plot of 51 SNPs in *ATM* genotyped in 92 controls and included in our study. Red: D´=1 and LOD ≥ 2. Blue: D´=1 and LOD < 2. White: D´< 1 and LOD < 2.


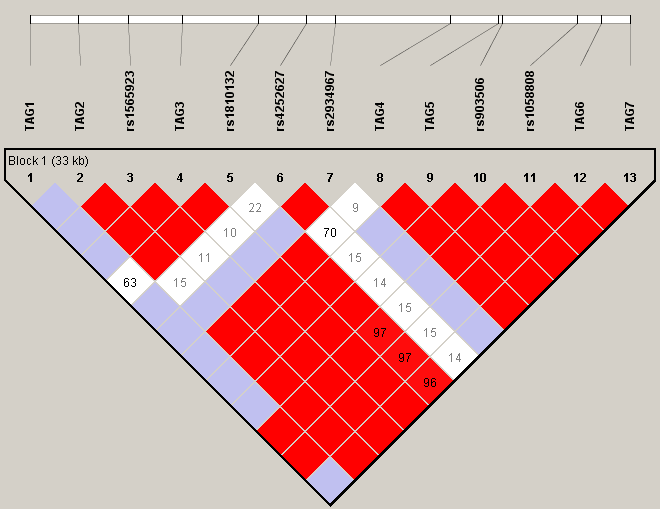


Supplementary Figure 2. LD plot of 13 SNPs in *ERBB2* genotyped in 92 controls and included in our study. Red: D´=1 and LOD ≥ 2. Blue: D´=1 and LOD < 2. White: D´< 1 and LOD < 2.

Supplementary Table 4. Global *P-*values for association of *ATM, CHEK2* and *ERBB2* haplotypes with tumour characteristic-defined breast cancer.

|  |  | Global *P*-values^a^ | | | | |
| --- | --- | --- | --- | --- | --- | --- |
| Tumour characteristic |  | *ATM*^b^ |  | *CHEK2*^c^ |  | *ERBB2*^d^ |
| TNM stage |  |  |  |  |  |  |
| 1 |  | 0.696 |  | 0.752 |  | 0.694 |
| 2 |  | 0.762 |  | 0.067 |  | 0.495 |
| 3-4 |  | 0.738 |  | 0.591 |  | 0.488 |
| Tumour size (mm) |  |  |  |  |  |  |
| ≤ 20 |  | 0.512 |  | 0.692 |  | 0.907 |
| 21-30 |  | 0.821 |  | 0.295 |  | 0.980 |
| >30 |  | 0.672 |  | 0.127 |  | 0.306 |
| Lymph node involvement |  |  |  |  |  |  |
| No |  | 0.513 |  | 0.653 |  | 0.818 |
| Yes |  | 0.967 |  | 0.138 |  | 0.483 |
| Grade |  |  |  |  |  |  |
| 1 |  | 0.232 |  | 0.837 |  | 0.819 |
| 2 |  | 0.635 |  | 0.959 |  | 0.828 |
| 3 |  | 0.842 |  | 0.433 |  | 0.729 |
| Oestrogen and progesterone  receptor status |  |  |  |  |  |  |
| ER+/PR+ |  | 0.397 |  | 0.594 |  | 0.506 |
| ER+/PR− |  | 0.654 |  | 0.154 |  | 0.566 |
| ER−/PR+ |  | 0.480 |  | 0.797 |  | 0.447 |
| ER−/PR− |  | 0.810 |  | 0.083 |  | 0.397 |
| Histological type |  |  |  |  |  |  |
| Ductal |  | 0.654 |  | 0.641 |  | 0.645 |
| Lobular |  | 0.702 |  | 0.209 |  | 0.968 |
| S-phase fraction |  |  |  |  |  |  |
| Low |  | 0.494 |  | 0.232 |  | 0.996 |
| High |  | 0.821 |  | 0.604 |  | 0.402 |

^a^ Cases divided in groups by their tumour characteristics and each group contrasted against all controls.

^b^ Likelihood ratio test with 6 degrees of freedom. Models include 5 common haplotypes and the 11 rare haplotypes combined into a single variable. The most common haplotype is reference.

^c^ Likelihood ratio test with 7 degrees of freedom. Models include 6 common haplotypes and the 19 rare haplotypes combined into a single variable. The most common haplotype is reference.

^d^ Likelihood ratio test with 8 degrees of freedom. Models include 7 common haplotypes and the 19 rare haplotypes combined into a single variable. The most common haplotype is reference.

Supplementary Table 5. Association of the tagSNPs in *ATM* and *ERBB2* with breast cancer risk.

| SNP ID | Number of  cases/controls | OR (95% CI)^b^ |
| --- | --- | --- |
|  |  |  |
| *ATM* |  |  |
| TAG1 | 1220/1440 | 1.05 (0.84-1.31) |
| TAG2 | 1119/1318 | 1.08 (0.93-1.27) |
| TAG3 | 1144/1346 | 0.89 (0.63-1.26) |
| TAG4 | 1538/1500 | 1.08 (0.94-1.24) |
| TAG5 | 1546/1493 | 1.00 (0.79-1.28) |
| TAG6 | 1152/1350 | 0.99 (0.88-1.12) |
| TAG7 | 1227/1408 | 1.01 (0.91-1.13) |
|  |  |  |
| *ERBB2* |  |  |
| TAG1 | 1494/1458 | 0.98 (0.88-1.09) |
| TAG2 | 1530/1481 | 0.90 (0.78-1.05) |
| TAG3 | 1459/1407 | 0.99 (0.88-1.11) |
| TAG4 | 1546/1481 | 0.97 (0.87-1.09) |
| TAG5 | 1548/1485 | 1.01 (0.91-1.13) |
| TAG6 | 1527/1486 | 1.00 (0.80-1.25) |
| TAG7 | 1532/1478 | 1.01 (0.90-1.12) |

^a^ In the controls.

^b^ Odds ratios are assessed assuming co-dominance and show the increase/decrease in breast cancer risk with each addition of the rare allele. Analyses were adjusted for age (5 year age-groups).

Supplementary Table 6. Common tagSNP haplotypes in *ATM* and *ERBB2* in relation to breast cancer risk.

|  |  | Haplotype proportions | |  |
| --- | --- | --- | --- | --- |
| Haplotype no. | Haplotypes | Cases | Controls | OR (95% CI)^b^ |
|  |  |  |  |  |
| *ATM* |  | (n = 1574^a^) | (n = 1513^a^) |  |
| 1 | AACGCCT | 0.414 | 0.408 | 1.00 (Reference) |
| 2 | AACGCTC | 0.231 | 0.231 | 0.99 (0.86-1.13) |
| 3 | AGCACCC | 0.150 | 0.139 | 1.06 (0.91-1.24) |
| 4 | AACGCCC | 0.062 | 0.076 | 0.81 (0.64-1.02) |
| 5 | TACGCCT | 0.064 | 0.061 | 1.03 (0.81-1.30) |
| 6 | AACGGTC | 0.043 | 0.043 | 0.97 (0.75-1.25) |
|  | Rare^c^ | 0.037 | 0.042 | 0.88 (0.66-1.16) |
| Global *P*-value^d^ | |  |  | 0.50 |
|  |  |  |  |  |
| *ERBB2* |  | (n = 1579^a^) | (n = 1516^a^) |  |
| 1 | GGCGACT | 0.296 | 0.295 | 1.00 (Reference) |
| 2 | AGTAACG | 0.166 | 0.165 | 1.01 (0.86-1.18) |
| 3 | GGCGGCG | 0.135 | 0.128 | 1.04 (0.88-1.23) |
| 4 | GACGACG | 0.116 | 0.128 | 0.91 (0.77-1.08) |
| 5 | AGTAGCG | 0.075 | 0.077 | 0.97 (0.79-1.20) |
| 6 | AGCAACG | 0.068 | 0.071 | 0.94 (0.76-1.17) |
| 7 | GGCGACG | 0.079 | 0.069 | 1.14 (0.91-1.41) |
| 8 | GGCGGTG | 0.048 | 0.051 | 0.94 (0.74-1.20) |
|  | Rare^e^ | 0.018 | 0.015 | 1.18 (0.77-1.81) |
| Global *P*-value^d^ | |  |  | 0.76 |

^a^ Information on at least 1 out of 7 tagSNPs

^b^ Analyses were adjusted for age (5 year age-groups).

^c^ 11 rare haplotypes combined. Each haplotype has frequency below 3% among the controls.

^d^ Likelihood ratio test.

^e^ 19 rare haplotypes combined. Each haplotype has frequency below 3% among the controls.

Supplementary Table 7. Association of *ATM* and *ERBB2* haplotypes with breast cancer risk, by breast cancer risk factors.

| Characteristic |  | *ATM* |  | *ERBB2* |
| --- | --- | --- | --- | --- |
| Age at menarche (years) |  | Global *P-*value^a^ |  | Global *P-*value^b^ |
| ≤12 |  | 0.189 |  | 0.664 |
| >12-14 |  | 0.109 |  | 0.950 |
| >14 |  | 0.548 |  | 0.083 |
| Age at menopause (years) |  |  |  |  |
| <49 |  | 0.817 |  | 0.963 |
| 49-52 |  | 0.272 |  | 0.586 |
| >52 |  | 0.841 |  | 0.317 |
| Age at first birth (years) |  |  |  |  |
| ≤24 |  | 0.803 |  | 0.646 |
| 25-29 |  | 0.185 |  | 0.920 |
| ≥30 |  | 0.984 |  | 0.914 |
| Parity |  |  |  |  |
| Nulliparous |  | 0.634 |  | 0.235 |
| 1 child |  | 0.931 |  | 0.723 |
| 2 children |  | 0.754 |  | 0.713 |
| ≥3 children |  | 0.832 |  | 0.236 |
| Body mass index (kg/m2) |  |  |  |  |
| <25 |  | 0.226 |  | 0.903 |
| 25-<28 |  | 0.427 |  | 0.199 |
| ≥28 |  | 0.803 |  | 0.086 |
| Family history^c^ |  |  |  |  |
| No |  | 0.566 |  | 0.504 |
| Yes |  | 0.664 |  | 0.214 |
| Duration of menopausal  hormone use (years) |  |  |  |  |
| Never |  | 0.820 |  | 0.473 |
| Oestrogen only |  |  |  |  |
| <4 |  | 0.448 |  | 0.335 |
| ≥4 |  | 0.202 |  | 0.291 |
| Oestrogen + progestin |  |  |  |  |
| <4 |  | 0.995 |  | 0.459 |
| ≥4 |  | 0.659 |  | 0.125 |
| Self-reported diabetes mellitus |  |  |  |  |
| No |  | 0.517 |  | 0.683 |
| Yes |  | 0.454 |  | 0.407 |

^a^ Likelihood ratio test with 6 degrees of freedom. Models include 5 common haplotypes and the 11 rare haplotypes combined into a single variable. The most common haplotype is reference.

^b^ Likelihood ratio test with 8 degrees of freedom. Models include 7 common haplotypes and the 19 rare haplotypes combined into a single variable. The most common haplotype is reference.

^c^ At least one 1^st^ degree relative with breast cancer.
